# What should progress in response-shift research look like?

**DOI:** 10.1007/s11136-021-02962-7

**Published:** 2021-07-30

**Authors:** Bruce D. Rapkin, Carolyn E. Schwartz

**Affiliations:** 1 Division of Community Collaboration & Implementation Science, Department of Epidemiology and Population Health, Albert Einstein College of Medicine, Bronx, NY USA; 2 DeltaQuest Foundation, Inc., 31 Mitchell Road, Concord, MA 01742 USA; 3 Departments of Medicine and Orthopaedic Surgery, Tufts University Medical School, Boston, MA USA

We are pleased that the interest in response shift continues to remain strong and we appreciate the In-Sync Group’s intentions to assess the field. Clearly, there is a growing consensus among researchers that response-shift phenomena are central to any understanding of quality of life (QOL). However, as this series of papers clearly states, much of the uncertainty about how to align and reconcile different definitions and methods remains unclear [1–4]. Twenty-five years after the initial work on response shift [5], it feels less than satisfactory to leave these as unresolved. As the In-Sync group argues, for progress to be made in the study of change in QOL, it will be necessary to specify and test falsifiable hypotheses about the nature of response shift.

In 2004, we set out to bring clarity to theory and measurement by strictly defining response shift in terms of changes in appraisal [6, 7]. The In-Sync papers each suggest ways to articulate and interpret key questions in QOL research and in healthcare decision-making in terms of response shift. We believe that applying the definition of response shift formalized by our appraisal model provides a way to specify hypotheses about divergences and convergences among the many different conceptual and methodological approaches that are discussed in these papers.

In the appraisal paradigm, QOL can only be understood by encompassing each individual’s unique point of view. Response shift represents a change in individual perspective—distinct from change in level of QOL but nonetheless meaningful. This perspective has led to a substantial body of work, including a *psychological theory* [6, 7] whose constructs have been examined in over two dozen peer-reviewed papers on multiple patient populations; a series of *measures* honed with data on over 6000 patients [8–10]; a *measurement theory* [10, 11] that accounts for the contextual nature of appraisal change; and a path for *intervention* [12].

It is important to clarify a misconception that was evident in the In-Sync work. Rather than being an alternative theory of how response shift can occur, appraisal is fundamental to defining and operationalizing recalibration, reprioritization, and reconceptualization response shifts. That is, you cannot have *re*-conceptualization without having conceptualization. Accordingly, response shift is an emergent phenomenon that can only be inferred when discrepant changes in QOL scores are explained by changes in appraisal. Such discrepant changes have most often been defined statistically as residuals but may also be based on comparison of individuals’ changes in self-reported QOL scores with clinician or proxy ratings of change, estimates based on observable performance, comparison to population-based norms, and even the then-test. However, residuals or other discrepancy scores are not in and of themselves measures of response shift. It is only when changes in appraisal explain these discrepancies that response shift can be inferred.

In order to advance work in this area, testable hypotheses about response shift must have clear theory. The appraisal model offers testable pathways to explain the unexpected impact of changes in health states and other life events. Influences of demographic, clinical and psychosocial antecedents listed in Vanier et al.’s [2] table of related domains are also expected to be mediated by appraisal.

The Sebille et al. [1] paper contrasts a number of statistical methods aimed at estimating or detecting response-shift effects from QOL data alone with those that directly measure psychological change. It is confusing that Sebille et al. have relegated the direct measurement of appraisal to being a “design method” rather than recognizing its centrality to any understanding of response shift. It should be noted that statistical approaches to detect response shift *essentially infer cognitive processes* of recalibration, reconceptualization, or reprioritization. As such, these statistical methods can best be validated against appraisal measures.

Both the Vanier et al. and Sawatzky et al. papers promote a perspective that response shift is a form of bias in measurement [2, 3]. This position is difficult to reconcile with an understanding of response shift as meaningful change. In the context of healthcare decision-making, providers discussing patient-reported QOL do not have a privileged perspective to correct patients’ “bias.” Rather, patients often legitimately see their QOL differently than providers, family members, or other caregivers do. Indeed, all observers have their own subjective perspectives. Articulating these perspectives with appraisal measurement can be useful for addressing cultural and racial tensions that influence outcomes in medicine.

At this point, response-shift theory has to go beyond reshuffling the deck. The In-Sync group has continued an ongoing dialogue about the importance of work that is grounded in testable theory. However measured, there needs to be a consensus that response shift is not bias. Rather it is about meaningful changes in individual perspectives that occur in reaction to changing health. The appraisal model directly takes hold of those fundamental cognitive processes. That is why we emphasize its centrality for this area of research. As we invited in our 2019 paper [13] in this journal, we strongly encourage work in the field that builds upon, improves upon, *or* substantively refutes work on appraisal. Work that further addresses appraisal or provides a more compelling and empirically grounded alternative to current appraisal theory is needed to advance and unify our field.

## Data Availability

Not applicable.
